# Biomimetic Coatings in Implant Dentistry: A Quick Update

**DOI:** 10.3390/jfb15010015

**Published:** 2023-12-30

**Authors:** Mohammed Aso Abdulghafor, Mohammed Khalid Mahmood, Herve Tassery, Delphine Tardivo, Arthur Falguiere, Romain Lan

**Affiliations:** 1 College of Dentistry, University of Sulaimani, Sulaimani 46001, Kurdistan, Iraq; 2 Faculty of Dentistry, Aix-Marseille University, CNRS, EFS, ADES, 13284 Marseille, France; delphine.tardivo@uniiv-amu.fr; 3 College of Dentistry, The American University of Iraq, Sulaimani 46001, Kurdistan, Iraq; 4 LBN Laboratory, 34070 Montpellier, France; 5 Oral Surgery Department, Timone Hospital, Aix-Marseille University, APHM, 13284 Marseille, France; 6 Oral Surgery Department, Timone Hospital, Aix-Marseille University, APHM, CNRS, EFS, ADES, 13284 Marseille, France; romain.lan@univ-amu.fr

**Keywords:** dental implants, coatings, surface modifications, antibacterial, osseointegration

## Abstract

Biomimetic dental implants are regarded as one of the recent clinical advancements in implant surface modification. Coatings with varying thicknesses and roughness may affect the dental implant surface’s chemical inertness, cell adhesion, and antibacterial characteristics. Different surface coatings and mechanical surface changes have been studied to improve osseointegration and decrease peri-implantitis. The surface medication increases surface energy, leading to enhanced cell proliferation and growth factors, and, consequently, to a rise in the osseointegration process. This review provides a comprehensive update on the numerous biomimetic coatings used to improve the surface characteristics of dental implants and their applications in two main categories: coating to improve osseointegration, including the hydroxyapatite layer and nanocomposites, growth factors (BMPs, PDGF, FGF), and extracellular matrix (collagen, elastin, fibronectin, chondroitin sulfate, hyaluronan, and other proteoglycans), and coatings for anti-bacterial performance, covering drug-coated dental implants (antibiotic, statin, and bisphosphonate), antimicrobial peptide coating (GL13K and human beta defensins), polysaccharide antibacterial coatings (natural chitosan and its coupling agents) and metal elements (silver, zinc, and copper).

## 1. Introduction

Nowadays, one of the most popular treatment choices for partially and completely edentulous arches is dental implants [1]. The intervention with implants promised greater outcomes than the treatment with traditional dentures, with an emphasis on alveolar bone preservation, esthetics, and prosthesis durability [2]. Osseointegration is essential to dental implant success, because it requires direct contact and interface between the peri-implant tissues and the implant surface in the absence of connective-tissue-layer involvement [3]. Osseointegration is also crucial for the success of bone grafts. The implant material’s biocompatibility, the surface and design characteristics of the implant, both macroscopic and microscopic, the quantity and quality of bone, an unhindered healing phase, the loading circumstances, and the implant finish are all important factors in achieving successful osseointegration of the implant [4,5].

Failure of dental implants can occur for a variety of causes. These include issues relating to implants, clinicians, and patients; also, infections and foreign-body responses may contribute to the quick loss of alveolar bone [6]. The loss of alveolar bone, which is typically accompanied by the deposition of microbial plaque and bacterial infections known as peri-implantitis, is the most frequent cause of implant failure [7,8].

**Figure 1:**
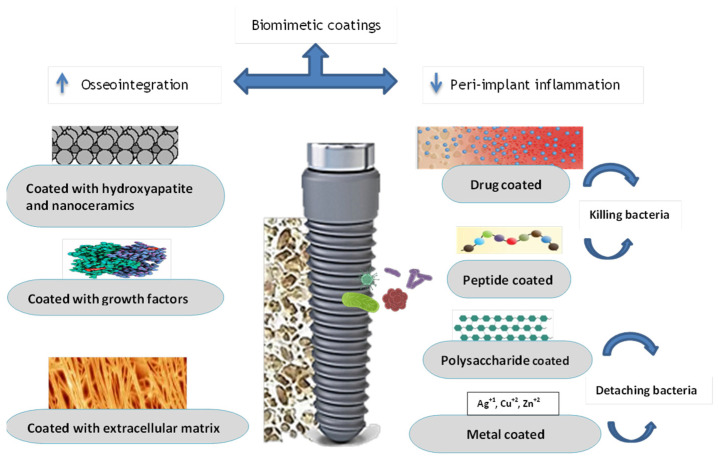
Overview of the review.

For dental implants to be clinically effective in the long term, osseointegration and the contact between the implant material and the bone are essential [9]. There is considerable evidence that improving osseointegration is strongly associated with the durability and prolonged clinical success of dental implants, as demonstrated in the research study [10]. A variety of surface coatings [11] and mechanical surface changes [12] have been studied to see whether they can improve osseointegration and bone formation. Surface medication leads to enhanced cell proliferation and growth factors, which results in an increase in the osseointegration process [13]. According to studies, the surface area of the implant increases with increased surface roughness, which leads to higher cell proliferation and cell growth [14]. Because of these surface modifications, the biocompatibility of the implant material is greatly improved, as is the adsorption of protein and cells [15]. This results in faster osseointegration and also a shorter period of healing, which is desirable for both clinicians and patients, and thereby improves the patient’s quality of life [5,9]. Hence, biomimetic coating is the process of dental-implant surface modification mainly targeting increasing osseointegration and reducing microbial biofilm formation. The aim of this narrative review is to provide a succinct update on the numerous biomimetic coatings that are used to improve the surface characteristics of dental implants, as well as their applications. Figure 1 shows the overview of this review.

## 2. Implant Surface Modifications

Metals are not new in dentistry. However, metals are materials with no biological activity, which contributes to their limited attractiveness as biomaterials [16]. During the last three decades, remarkable technological breakthroughs in ceramics and polymers have permitted their usage in medical devices. Due to their superior biofunctional properties, ceramics and polymers have replaced many metal-based devices [17]. Due to their strength, durability, and lifespan, metals still account for over 70% of implant devices in the medical and dental industries [18]. Ceramics and polymers cannot replace metallic biomaterials. Metals cannot have biofunctions added to them during production procedures such heat treatment, casting, melting, or forging [19].

**Figure 2:**
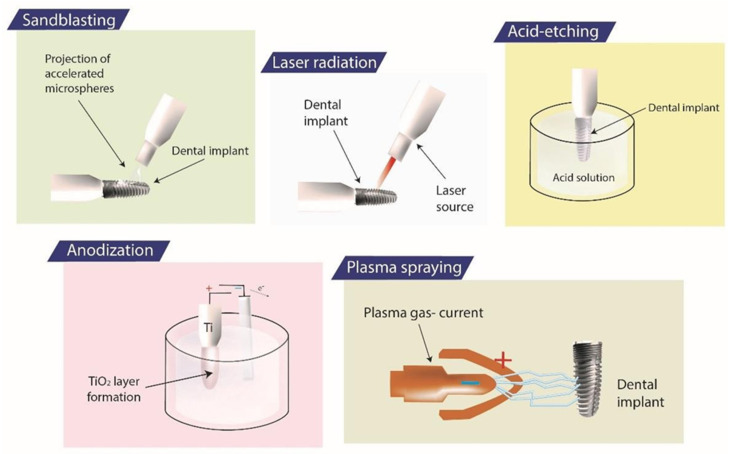
Different techniques of dental-implant surface modifications. Reprinted from Ref. [24].

The surface modification (Table 1) has an impact on the material’s surface shape, structure, and composition, while keeping its core mechanical properties. Metals with biofunctional characteristics have also been required recently [20]. Dental implants must be compatible with hard and soft tissue for osseointegration and bone development, and antibacterial characteristics to prevent biofilm formation. These biofunctional properties oppose protein adsorption and cell adhesion [21]. A metallic implant reacts with living tissues right away when it is placed into a human body. Put differently, the biofunction of a metallic substance is determined and characterized by its early reaction. Surface biofunction can be enhanced by surface alteration. For these reasons, many methods of metal surface modification are being researched and tested (Figure 2) [22,23,24]

## 3. Biomimetic Coating

Implant dentistry progress encourages the advancement in improving implants’ mechanical and biological properties. Aside from altered processes that cause the implant surface to distort plastically, certain supplementary technologies can produce surface depositions known as coatings [25]. The most essential way to improve clinical effectiveness is to update implant-coating technology. Various surface-coating combinations have been used to increase implant biocompatibility, bioactivity, and antibacterial potential [21]. Biomimetic dental implants are regarded as one of the most recent clinical advancements in implant surface modification. Otto Schmitt used the term “biomimetics” for the first time in the 1950s [26]. The structure, synthesis, and operation of biologically created materials, as well as biological systems and processes, are all included in biomimetics, which is the artificial emulation of natural processes to create similar products [27,28,29].

**Figure 3:**
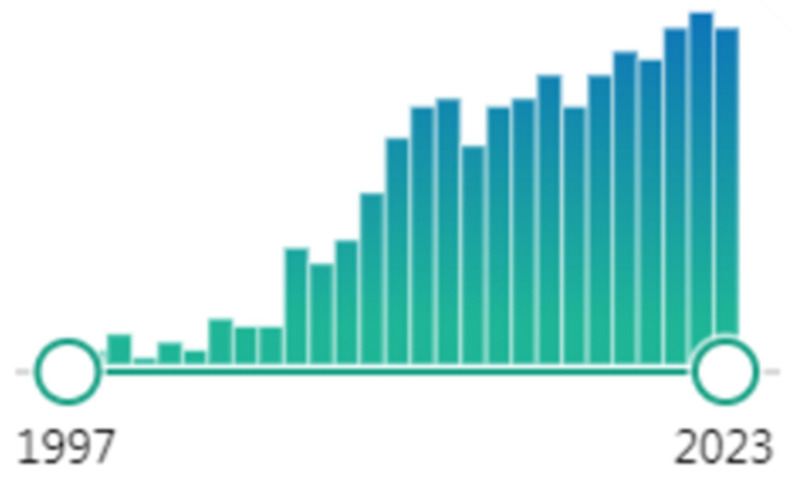
Trend in publications related to biomimetic dental implants, according to PubMed.

The ideal characteristics of biomimetic agents are discussed as the following: the ability to generate proper cell differentiation for promoting new bone formation, easy synthesis or manufacturing, with no extraction from allografts to reduce the danger of infectious–contagious disease transmission, resorbability in response to osteogenic activity, eliminating issues with implant loss owing to coating delamination, not generating immunological responses in the host, chemical stability until the implant is inserted into the surgical socket, and finally, a good cost-to-benefit ratio [29,30,31]. Table 2 shows the key properties of biomimetic coatings and Figure 3 illustrates the trend in publications related to biomimetic dental implants, according to PubMed.

### 3.1. Coating to Improve Osseointegration

**Figure 4:**
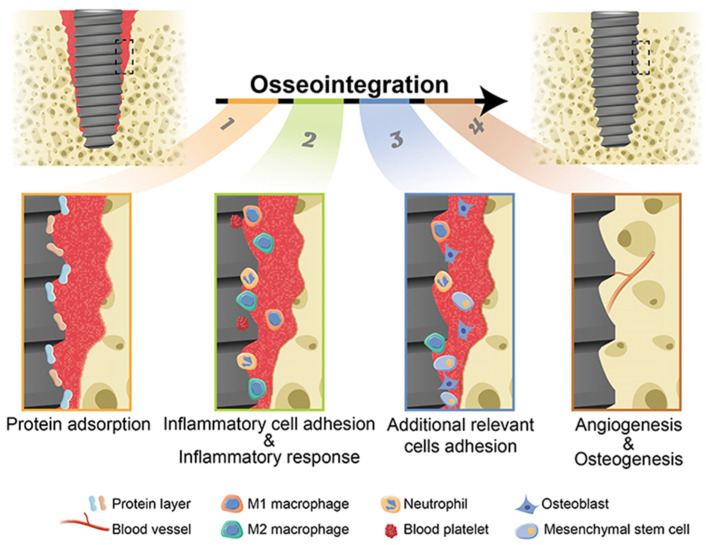
Stages of osseointegration around dental implants. Reprinted from Ref. [40].

Although advances in surface topography have resulted in improved osseointegration, a wide range of inorganic and organic coatings are being researched, to increase the tissue integration of dental implants [38]. The mechanical performance of strong biometals and the bone-bonding potential of bioactive materials can be combined when bioactive materials are coated on them. The surface features of dental implants, such as chemical inertness, cell adhesion, and antibacterial properties, can be impacted by coatings that differ in thickness and roughness [21,39]. The following section mostly discusses current advancements in dental implants coated with bioactive materials. Figure 4 shows the stages of osseointegration around dental implants [40].

#### 3.1.1. Hydroxyapatite Layer and Nanocomposites

Of the several methods, one of the most popular is coating implants with hydroxyapatite (HA) [41]. HA is a biologically stable type of calcium phosphate that mineralizes to strengthen the organic matrix without causing inflammation or immunogenicity [42]. It is composed of naturally occurring ions from physiological settings, and has good osteoconductive and osteointegration properties. Several ion-substituted hybrid anchors paved the path for implant architecture featuring diverse biological activities. Ion-substituted HA coatings have been demonstrated to significantly enhance cell attachment, despite the possibility that they will negatively affect the growth and differentiation of cells attached to the coating surface [43,44].

Moreover, the bioactivity and osteoconductivity of the titanium substrate can be enhanced by the HA layer. By using a micro-arc oxidation process to create a porous hydroxyapatite-coated titanium alloy surface, it will be possible to enhance the mechanical properties and promote bone formation by increasing the interface contact rate and bone-to-implant contact [45]. Recently, implant surface qualities have been enhanced by the use of nano-hydroxyapatite, which can be combined with collagen, bioglass, or titanium dioxide, to create a composite that mimics the bio-environment of natural bones [46]. The specific surface area and adsorption capacity of nano-sized particles are greatly increased. The nano-hydroxyapatite coating provides better bone bonding with dental implants over time, when compared to a standard dual acid-etched surface. Because HA coatings can immobilize growth factors and proteins through non-covalent interactions, hybrid coatings that hasten the healing of bones have been created [21].

In conclusion, since HA’s chemical and crystallographic structures are remarkably comparable to those of human bone, virtually all biocompatibility issues are resolved, making its application particularly promising. Nevertheless, HA has certain drawbacks, including fracture toughness, low tensile strength, and brittleness. However, when created as a coating for practical use, HA’s benefits can be fully utilized.

Table 3 contains a selection of a list of recent publications on nanoceramics used as coating materials.

#### 3.1.2. Growth Factors

Numerous growth factors are produced by platelets and macrophages, which are present during the first phase of osseointegration and help to initiate the second phase [57]. To speed up this process, coating materials containing Transforming Growth Factor (TGF), Platelet-Derived Growth Factor (PDGF), and Fibroblast Growth Factor (FGF) have been employed [20,58].

The signal protein known as vascular endothelial growth factor, or VEGF, is involved in both vasculogenesis and angiogenesis. It has been demonstrated that VEGF increases alkaline phosphatase (ALP) activity, activates genes and protein expression related to vasculogenesis, and increases primary rat osteoblast proliferation in vitro. In the related in vivo experiment, coating the implant with VEGF significantly increases the activation of osteoblasts and endothelial cells [59,60]. When compared to SiHA or VEGF-coated groups, respectively, the silicon substituted hydroxyapatite (SiHA)-coated scaffolds combined with VEGF had a synergistic effect on enhanced ossification, larger bone trabeculae, and greater angiogenesis degree in a sheep model [61].

Growth factors known as bone morphogenetic proteins (BMPs) are crucial for the development of cartilage and bone. BMPs have the ability to regulate osteogenic cells and promote bone mesenchymal stem cell (MSC) development [62,63]. More encouraging findings are achieved when BMPs are used, since they have a strong biological potential for osteoinduction. BMPs (BMP-2, BMP-4, and BMP-7) are members of the superfamily of Transforming Growth Factors (TGF) [64]. When compared to anodized implants, titanium implants containing BMP-2 had better bone-to-implant contact, more new bone development, and a higher density of surrounding bone than acid-etched implants [65,66]. BMP-7 has shown promise as a bone regeneration stimulant throughout the years. Research has demonstrated that administering a comparatively modest concentration of BMP-7 locally can enhance osseointegration through the development of a particular delivery mechanism including a titanium surface covered with poly-ethyl acrylate [67,68].

To conclude, growth factors have been shown to improve bone regeneration and osseointegration. The initial findings reported in the literature appear encouraging, even if further clinical research is needed to confirm the long-term benefits of growth factors as dental implant surface coatings. Table 4 shows a selected list of recent articles on the surface implant coating with growth factors.

#### 3.1.3. Extra Cellular Matrix

Another method to increase dental implant biocompatibility is by accumulating extracellular matrix (ECM) proteins on implant surfaces, which control cell-matrix adhesion [74]. Fibroblast growth factors stimulate fibroblasts to secrete extracellular matrix (ECM) proteins like hyaluronan, collagen, chondroitin sulfate, fibronectin, and elastin during the proliferative stage of osseointegration [57]. By rearranging intracellular microfilaments and microtubules, these extracellular matrix proteins seem to be essential for the early stages of bone healing. They also help cells adhere and spread, and via the action of cell surface integrins and fibronectin arginine–glycine–asparginine motifs, they guide osteoprogenitor cell migration to the implant surface [75,76]. However, using such a unique protein has three significant limitations: the high cost of synthesis, the molecule’s antigenicity and instability, and the macromolecule’s steric hindrance in focal adhesion [77,78].

There is enough evidence from the literature on the usefulness and contribution of ECMs as coating materials to the osseointegration process. However, in order to allow the use of early loading techniques and ensure implant success in patients with damaged bone tissue, future research should look into the developments in dental implant surface design including ECMs. These developments are critical to improve the healing process and enhancing bone formation. Table 5 contains a selection of recent articles on the surface coating with ECM.

### 3.2. The Antibacterial Performance of Coating

**Figure 5:**
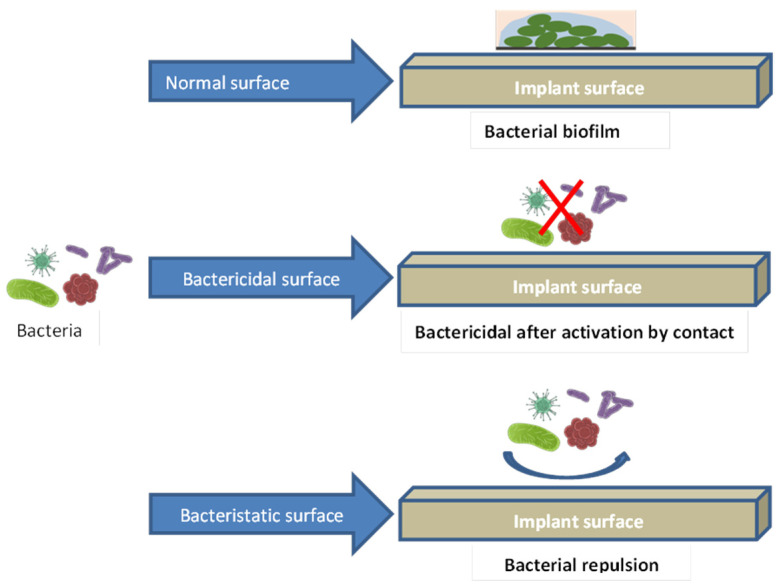
Bacterial interaction with naked implant surface, a bactericidal surface, and a bacteriostatic surface. The red × in the second diagram indicates bactericidal killing bacteria.

Implant-associated infections are a frequent postoperative outcome of implant rehabilitation, which can cause patient discontent, additional costs, and, potentially, implant failure. Microorganisms within implants are shielded from antibodies by biofilms adhering to the implant surface [88,89]. On the other hand, antibiotic overuse can potentially increase the spread of drug-resistant microorganisms [90]. In an effort to combat this, a large number of researchers have worked to create specialized implants with functional coatings that can either specifically target and kill the bacteria or prevent bacterial adherence and biofilm formation [91,92]. Figure 5 shows the bacterial interaction with naked implant surface, a bactericidal surface, and a bacteriostatic surface.

#### 3.2.1. Drug-Coated Dental Implant

Antibiotics, Simvastatin, and bisphosphonate are examples of drug coating on the surface of dental implants [93]. Antimicrobial surfaces have been developed in dental implantology in two different ways, so far. Antimicrobials are actively released from type I surfaces to inhibit bacterial adherence and promote killing, whereas antimicrobials are permanently bound to type II surfaces, to prevent long-term bacterial adhesion and promote killing [94,95]. Implants featuring a type I surface have proven effective in treating infections related to implants; however, their eluting activities may pose challenges, as the initial burst of antibiotics happens during the first week of implantation and then decreases exponentially over time, raising the possibility of generating bacteria resistant to antibiotics that will persist for a long time [96,97].

A number of bactericidal and bacteriostatic chemicals were permanently added to implant surfaces, in order to generate a type II surface that prevents the formation of biofilm around dental implants, in order to solve this restriction of the type I surface [88,98]. For instance, applying a bacteriostatic medication like tetracycline permanently on implant surfaces efficiently eliminated bacteria that may otherwise infect the implant surface, promoting cell proliferation and bone healing [99]. In a similar way, prolonged vancomycin coating of titanium implants inhibited *Streptococcus aureus* colonization, while speeding up bone repair. [100]. Vancomycin therefore works better than antibiotics that cling to germs permanently, such as gentamicin, which becomes ineffective when bacteria are re-exposed [98,101]. However, widespread use of vancomycin would exacerbate the worry about the spread of vancomycin-intermediate and vancomycin-resistant *Streptococcus aureus* (VISA) strains [102]. Bisphosphonates (alendronate, etidronate, tiludronate, and zoledronate) are medications that stimulate osteoblasts and bone production, while blocking osteoclastic activity and bone resorption [103,104]. For the antibacterial activity, bisphosphonate-coated implants revealed a significant decrease in bacterial adhesion [105]. Simvastatin may promote bone growth and enhance bone-to-implant contact by upregulating the production of VEGF and BMP [106]. Porous titanium surfaces coated with simvastatin improved alkaline phosphatase activity, type I collagen synthesis, and osteocalcin release from pre-osteoblasts, in vitro [107]. In addition, Simvastatin-Hydroxyapatite coatings are reported to have good antibacterial performance [108].

To sum up, in order to provide targeted drug delivery and therapeutic activities, a variety of strategies have been used to induce prolonged drug release from dental implants, for a range of medications. Drug excretion from dental implants has been documented in a number of in vitro and in vivo animal models, and in experimental investigations; nevertheless, more clinical study is necessary before considering drug-eluting dental implants for clinical application.

#### 3.2.2. Antimicrobial Peptide Coating

Innate host defense antimicrobial peptide coatings (AMPs), which are tiny cationic peptides, demonstrate a wide range of antibacterial activity against different pathogens, such as gram-positive and gram-negative bacteria, and they also lessen the development of bacterial resistance [109,110]. Applications for AMPs are numerous; however, one of the more notable uses is the covalent immobilization method used to biofunctionalize titanium to confer antibacterial properties [111]. To stop germs from growing on implant surfaces, a number of bactericidal peptides have been employed, such as GL13K and human beta defensins (HBDs) [112]. The salivary defense protein BPI fold-containing family A member 2 (BPIFA2) is the source of GL13K, a parotid secretory protein [113]. According to research by Holmberg et al., GL13K applied to implant surfaces has a bactericidal effect against *Porphyromonas gingivalis*, while preserving cytocompatibility and promoting sufficient proliferation of osteoblasts and gingival fibroblasts [114]. Likewise, HBDs showed broad-spectrum antibacterial action, and stimulated osteoblast and mesenchymal stem cell growth when applied to implant surfaces [115,116].

Peptide antibiotics are gaining traction as a viable implant coating material to lessen/prevent peri-implantitis and increase dental-implant success rates, because of their wide range of activity and less potential to cause bacterial resistance. It would facilitate their transition to clinical application if more evidence could be provided about the reliability of these peptides adsorbed onto implant surfaces and their resistance to buffers, pH changes and bodily fluids.

#### 3.2.3. Polysaccharide Antibacterial Coating

Natura Chitosan is a neutral cationic polymer generated from the deacetylation of chitin [117]. Chitosan-immobilized implant surfaces have been shown to exhibit antibacterial characteristics [118,119]. Triethoxysilylpropyl succinic anhydride (TESPSA) functions as a coupling agent that has the potential to form a stable double-peptide bond with chitosan [120]. The TESPSA/chitosan coating demonstrated good adhesion resistance at titanium surfaces, according to Campos et al. [121]. In order to boost antibacterial activity, Palla-Rubio et al. incorporated silica–chitosan hybrid materials onto titanium implants. They found that a suitable concentration of 5–10% for encapsulated chitosan displayed antibacterial features [122]. The antibacterial properties of polyelectrolyte multilayers containing hyaluronic acid and chitosan were also appreciated against *S. aureus* [123]. Ag-conjugated chitosan nanoparticle coating on titanium surface shows promise in preventing growth of *S. mutans* and *P. gingivalis* and in reducing the formation of biofilms and bacterial adhesion [124,125]. Additionally, antimicrobials may be provided by using chitosan coatings in a biocompatible way, to prevent the growth of bacteria. On the titanium surface, chitosan coatings containing either 0.2% or 20% tetracycline digluconate were applied and tested for their ability to fend off infections such *Actinobacillus*, *Actinomycetemcomitans*, and *Staphylococcus epidermidis*. A total of 89 percent of tetracycline and 100 percent of chlorhexidine were released by the coatings in 7 and 2 days, respectively; nevertheless, the chlorhexidine that was released posed a risk to human osteoblasts and fibroblasts [126].

In summary, considerable progress has been made in using polysaccharides as coatings with antibacterial properties. A wide variety of the most often utilized coating processes have been developed, each with varying possibilities for development. Future technological developments could focus on improving the structures and properties of polysaccharides as coatings, as well as creating commercially viable polysaccharide-based coatings for particular uses.

#### 3.2.4. Antibacterial Properties of Metal-Element Components

Antibiotic resistance and a restricted antibacterial range are just two of the drawbacks associated with using antibiotic coatings on dental implants [127]. Silver, copper and zinc have also been used in coating of implants as an alternative, because of their antibacterial properties and the fact that they are available in nanoparticulate forms [128,129]. Silver’s multilayer antibacterial action is widely recognized, and assures a broad range of antibacterial activity, as well as long-term antibacterial activity [130]. Table 6 shows several elements that were used as coating that have a good antibacterial property.

Antibacterial metal alloys exhibit strong and durable antibacterial capabilities, together with excellent mechanical properties, corrosion resistance, and all-round qualities that point to their possible future applications. Nonetheless, a few issues remain prior to the clinical application, such as the exact antibacterial mechanism and possible toxicity of some elements.

## 4. Conclusions

Biomolecules, such as bioactive chemicals and multifunctional molecules, can be attached to implant surfaces to enhance the osteogenetic process around implants. This process includes inducing cell adhesion, providing an osteogenic stimulation, and potentially having antimicrobial properties.

To improve osseointegration, the use of hydroxyapatite and BMP in the coatings of implants appear to be the most promising methods. The treatment of implant surfaces with nano-hydroxyapatite, which can be combined with collagen, may create a composite mimicking the bio-environment of natural bones, and thus reduce inflammation and accelerate the healing of the peri-implant bone.

Growth factors such as VEGF, which is involved in angiogenesis, but especially BMPs, are crucial for increasing bone-implant contact and osteoinduction, allowing the development of new bone on the implant periphery.

The covering of implant surfaces with ECM proteins such as growth factors from fibroblasts appears essential at the early stages of bone healing, but presents significant limitations, such as their high synthesis cost, antigenicity and instability of the coating material.

Concerning the antibacterial performance, the addition of tetracycline or bisphosphonate to type II implant coatings to prevent bacterial adhesion and the formation of biofilms seems to be the method most often used.

Regarding AMPs to prevent germs from growing on implant surfaces, GL13K and HBDs showed broad-spectrum antibacterial action, while stimulating the growth of osteoblastic and mesenchymal stem cells.

Other antibacterial compounds, such as TEPSA, chitosan, or multilayer silver implant coatings have also reported good results, but should be developed particularly against the resistance or profiling of specific strains of the oral environment, such as *S.aureus*, *S. mutans* and *P. gingivalis*.

Therefore, long-term clinical studies are still necessary to assess the effectiveness of various coatings and ascertain the success rates of novel implant coatings, even in light of the encouraging results. In addition, more investigation is needed to ascertain whether standard implant surface treatments and coatings can yield dependable therapeutic outcomes, especially with regard to attaining osseointegration stability and preventing inflammation, mobility, infection and mechanical issues.

## Figures and Tables

**Table 1:** Different methods of surface modifications.

Physical Surface Modifications (Subtractive)	Chemical Surface Modifications (Additive)	Biological Surface Modifications (Biomimetic)
Plasma spraying Low-pressure plasma spraying High-velocity oxy-fuel spraying Sputter deposition Magnetron sputtering Ion beam-assisted sputtering Pulsed laser deposition	Sol–gel deposition Electrophoretic deposition Electrochemical deposition Acid etching Anodization Peroxidation Alkaline treatment Fluoride treatment Vacuum treatment Plasma coating	Extracellular matrix Peptides Growth factors (BMPs, PDGF, and FGF) Drugs (antibiotic, statin, and bisphosphonate)

**Table 2:** Key properties of biomimetic coatings.

Properties of Bioactive Coatings	Studies	Findings
Bioactivity and osseointegration	Mackovic et al., 2012 [32]	With their extremely quick kinetics for bone-like hydroxyapatite mineralization and non-toxic effects on osteoblast cells, nanoscaled bioactive glass particles are a potentially useful material for bone-tissue engineering.
	Li et al., 2018 [33]	Carbonated hydroxyapatite (CHA) bioceramic coating with synergistic surface chemistry and topography alteration has a bright future as an implant coating, to promote optimal osseointegration.
Cellular response	Yu and Wei, 2013 [34]	Cell adhesion on distinct biomaterial surfaces is directly influenced by substrate surface qualities, which in turn influence cell proliferation and differentiation.
Ion dissolution and osteogenesis	Wu et al., 2020 [35]	Researchers used biological coating and surface topography modification to make biomimetic titanium implants with good-quality osteogenic potential.
Mechanical performance	Sebdani and Fathi, 2011 [36]	The elastic modulus, hardness, and fracture toughness of produced composite coatings increased as forsterite concentrations rose.
	Erol-Tygun et al., 2013 [37]	Modified bioglasses (such as nanoparticles) may increase the mechanical characteristics of these materials (hardness, elastic modulus, and tensile strength).

**Table 3:** Surface coating of implants using nanoceramics.

Studies	Methodology	Findings
Ripamonti et al., 2012 [47]	Plasma sprayed with crystalline hydroxyapatite	The findings in nonhuman primates suggest that geometrically built plasma-sprayed titanium implants are intrinsically osteogenic, with the concavities creating an ideal microenvironment for inducing bone development.
Alghamdi et al., 2013 [11]	Calcium phosphate (CaP) coating sprayed by radio frequency magnetrons	In both healthy and osteoporotic situations, dental implants modified with a thin layer of calcium pseudophosphate (CaP) coating efficiently enhance osseointegration.
Jing et al., 2015 [45]	HA coating by micro-arc oxidation approach	Bone ingrowth and the strength of the bone–implant interface will be significantly improved by this coating process.
Carradò et al., 2017 [48]	Sodium titanate/hydroxyapatite nanoporous bilayer	Osteointegration and osteoconduction *in vivo* are enhanced by a nanoporous hydroxyapatite/sodium titanate bilayer. It avoids delamination during screwing and may strengthen the durability of HA-coated dental implants without adhesive failures.
Łukaszewska-Kuska et al., 2018 [49]	HA coating using a direct electrochemical method	Potential advantage in chemical and physical properties that promote osseointegration.
Hu et al., 2018 [50]	Nanostructured HA coating on Ti-6Al-4V implants	Ti-6Al-4V implants covered with nanostructured HA may enhance osteointegration in diabetes animals by increasing angiogenesis and osteogenesis and addressing pathological bone loss.
Fang et al., 2019 [51]	Nanocrystalline hydroxyapatites with SDF-1	Biomimetic HA microsphere can promote alveolar bone repair.
Eawsakul et al., 2020 [52]	Double layers of gold nanoparticles	The coating possessed homogeneity and good biocompatibility, promoted osteoblast cell proliferation and had good stability.
Yu et al., 2021 [53]	Polydopamine nanoparticles functionalized with hydroxyapatite (HA/nPDAs) coated in three dimensions on implant surfaces	The coating’s ability to prevent reactive oxygen species (ROS) and encourage osteogenesis in both normal and high ROS environments (like diabetes, periodontitis, and osteoporosis) showed great promise for enhancing implant osteointegration, particularly in situations where high ROS levels are brought on by diseases.
Su et al., 2022 [54]	Composite multifunctional coating of polydopa-mine/hydroxyapatite/gelatin (PHG) prepared using gelatin and polydopa-mine/hydroxyapatite nano-particles	The proposed PHG coating may increase soft tissue sealing and bone bonding.
Alcudia et al., 2022 [55]	Porous silver nanoparticle/polycaprolactone/polyvinyl alcohol coatings	This coatings exhibited excellent adherence and a honeycomb-like surface structure that could facilitate vascularization of the implant and improve osseointegration.
Mokobia et al., 2023 [56]	ZnO-NPs-Coated implants	Implant fixation was improved by ZnO-NPs coating on metal surfaces because it promoted osteogenesis and soft tissue integration. Furthermore, to achieve a strong biological attachment for implants, osteoconductive nanoparticles formed a chemical relationship with bone. There is little doubt that implants with ZnO-NPs placed to their surfaces exhibit superior clinical outcomes due to a decreased risk of infection.

**Table 4:** Surface implant coating with growth factors.

Studies	Methodology	Outcomes
Lee et al., 2010 [69]	Titanium implants covered with a biodegradable polymer and basic fibroblast growth factor (bFGF).	The study’s findings suggest that electrospraying polylactic-co-glycolic acid (PLGA) and beta-fibroblast growth factor (bFGF) onto a titanium implant may promote bone formation adjacent to the implant’s surface.
Kim et al., 2013 [66]	Anodized implants covered in a mixture of human BMP-2 recombinant and human VEGFs.	Encourage the growth of vertical alveolar bone, yet it is unknown how rhBMP-2 and rhVEGF work together.
Schliephake et al., 2015 [70]	Oligodeoxynucleotides (ODNs) were anchored to the surface of sandblasted acid-etched (SAE) titanium screw implants and were hybridized with complementary strands of ODN conjugated to rhVEGF165	Accelerate the bone-implant contact of titanium implants that have been sandblasted and etched to a certain point. The growth factor appears to have a limited effect on the tissue right next to the surface of the implant.
Guang et al., 2017 [59]	Coating the implant with VEGF in vivo	Experiments could help osteoblasts and endothelial cells grow.
Yang et al., 2017 [71]	Titanium disc and screw types coated with human bone morphogenetic protein-2 (hBMP-2) and human growth and differentiation factor-5 (hGDF-5) to allow for the controlled release of the growth factors.	Enhance the clinical characteristics of implants for use in dentistry and orthopedics.
Al-Jarsha et al., 2018 [68]	Poly-ethyl acrylate (PEA)-coated titanium discs were adsorbed with human bone morphogenetic protein 7 (BMP-7).	Cell adhesion, proliferation, mineralization, and the production of osteogenic markers (osteopontin and osteocalcin) demonstrated that, in the absence of PEA coatings, the system was more effective in promoting osteodifferentiation of mesenchymal cells than combinations of titanium and BMP-7.
Keceli et al., 2020 [72]	PDGF and BMP-6 are loaded into the titanium implant after anodization.	There is a considerable probability that the early osseointegration phase will be prolonged as a result of a more favorable factor release and its role in the mineralization, proliferation, and related gene expression in osteoblastic cells.
Eawsakul et al., 2021 [52]	Creating BMP-2 immobilization on titanium that has been altered using the layer-by-layer method (LBL).	Enhanced osteoblast cell proliferation and exhibited an increase in stability.
Palermo et al., 2022 [60]	Using concentrated growth factor (CGF) permeated dental implants.	Improved osseointegration and post-surgical problems.
Maekawa et al., 2022 [73]	The first study to use BMP gene delivery combined with chemical vapor deposition (CVD) technology on titanium to encourage in vivo bone-to-implant contact and repair.	Enhances alkaline phosphatase activity and osteoblast cell development in vitro; enhances alveolar bone regeneration and bone-to-implant contact in a manner akin to high exogenous BMP-7 dosages in vivo. This new method of targeted gene distribution on implant surfaces provides an alternative to alveolar bone rebuilding.

**Table 5:** Surface coating with ECM.

Studies	Methodology	Findings
Morra et al., 2010 [79]	Collagen’s biochemical surface alteration in reaction to acid-etched titanium surfaces.	Results suggest that surface topography (morphological) and surface linkage of bioactive chemicals (biochemical) signals might work in concert to produce multifunctional implant surfaces.
Alghamdi et al., 2013 [80]	Comparison of three types of implants: uncoated, nano-CaP-coated, and coated with type 1 collagen.	Results failed to demonstrate a consistent beneficial effect of the collagen covering on bone growth throughout a three-month period, following implantation.
Lee et al., 2014 [81]	The development of peri-implant bone in implant groups that were uncoated (UC) and coated with HA, collagen plus HA (CH), and collagen, HA, and bone morphogenetic protein-2 (BMP-2).	Compared to the other groups, the BIC and new bone formation were significantly higher in the CH group. There were no notable variations observed in the other groups.
Korn et al., 2014 [82]	Collagen was combined with sulfated hyaluronan (sHya) or chondroitin sulfate (CS) in the coatings.	Implant surface coatings made of the selected organic ECM components demonstrated some potential to affect in vivo osseointegration.
de Barros et al., 2015 [83]	The implant surfaces underwent sandblasting and acid etching, and a portion of them were also coated with chondroitin sulfate and collagen type II (collagen/CS).	The width of the peri-implant gap affects the formation of peri-implant bone. There was not enough newly formed bone to completely fill in all the gaps surrounding each surface. The coating had a beneficial effect on bone growth when it was close to the surface.
Raphel et al., 2016 [84]	Elastin-like protein (ELP) that undergoes chemical modification to allow for new photocrosslinking and solution processing techniques to create stable coatings on the surfaces of titanium-based orthopedic and dental implants.	ELP coatings facilitate early implant loading, and may lessen micromotion, which may lead to aseptic loosening and early implant failure. They are also resistant to surgical implantation and accelerate osseointegration.
Yin et al., 2019 [85]	TNS-MAP is the designation given to titanium that has been alkali-treated and has nanonetwork structures (TNSs) covered with mussel adhesive protein (MAP).	TNS-MAP, a novel biocomposite implant material, with potential applications in orthopedics and practical dentistry.
Wu et al., 2020 [35]	TiO_2_ nanotubes or sandblasting and acid etching the surface of titanium were used to modify it. Mineralized extracellular matrix (ECM) made from cultured bone-marrow mesenchymal stromal cells was then applied.	The results demonstrated a viable strategy for producing biomimetic titanium implants with good osteogenic capacity, by combining surface topographical alteration with biological coating.
Syam et al., 2021 [86]	Dip-coating titanium (IDCT-Ti) implants with tetrapeptide Gly-Arg-Gly-Asp (GRGD).	The topography, hemocompatibility, and wettability of the implant surface—all of which are linked to enhanced osteoblast-cell adherence to implant surfaces and osseointegration—were positively impacted.
Rappe et al., 2022 [87]	The metallic foams were treated with an inorganic alkali thermochemical process and grafted with a cell adhesive tripeptide (RGD), in order to create a bioactive surface.	Combining these two techniques may be beneficial in improving the stability and osteointegration of porous metallic implants.

**Table 6:** Surface coating with antimicrobial properties of metals.

Studies	Methodology	Findings
[131]	AgNPs with polydopamine (PDA) coating applied to titanium.	May successfully prevent the growth of microorganisms against *S. mutans* and *P. gingivalis.*
[132]	Spin-coating technology was used to manufacture a series of Zn-incorporated coatings on micro rough titanium (Micro-Ti) using the sol–gel process.	Encourages osseointegration and prevents gram-positive and gram-negative germs from adhering to surfaces.
[133]	A two-step hydrothermal process was used to create nanorod-array structured coatings with a controlled-release feature of zinc (Zn) based on the in situ conversion of ZnO to ZnO@ZnS. This method gave titanium surface cell selectivity.	Maintained a strong antimicrobial effect against *S. aureus* and *E. coli*
[134]	Zinc ions and fluoride integrated into calcium phosphate coatings.	Possess bactericidal effects, particularly efficient at preventing the proliferation, colonization, and adherence of *P. gingivalis.*
[135]	TiOB^®^ (chemically oxidized titanium) coating containing ionic zinc.	Revealed that TiOB^®^ functionalization with ionic zinc demonstrates bactericidal characteristics similar to a coating containing gentamicin.
[136]	Zinc oxide (ZnO) nanoparticles.	Displayed antimicrobial properties
[137]	Copper nanoparticles (CuNPs).	Can release copper ions, which are thought to have a dual function in aiding in the development of new bone and avoiding infection.
[138]	Calcium silicate coatings containing cerium oxide (CeO_2_-CS).	Promoted osteoblast differentiation, demonstrated significant antibacterial efficacy against *E. faecalis* while maintaining acceptable biocompatibility.
[139]	Tantalum-based implant.	Coated surface performed significant antibacterial action against *F. nucleatum* and *P. gingivalis.*
[140]	Poly (lactic-co-glycolic acid)/Ag/ZnO nanorods coating.	Provided a strong antibacterial activity and high degree of cytocompatibility.
[141]	Using plasma electrolytic oxidation (PEO), selective laser melting (SLM) produced volume-porous Ti-Ta-Nb-Zr scaffolds with a surface biofunctionalized.	Provided robust osteogenic stimulation and antimicrobial activity, without causing cytotoxicity in mammalian cells.
[142]	Silver/strontium glass integrated polyelectrolyte multilayer coatings on 316L stainless steel.	Angiogenesis, osseointegration, and antibacterial activity were all improved by the PEM/AgSrMBG coating’s prolonged release of silver and strontium ions.
[143]	Titanium substrates were treated with phosphorus, calcium, and copper co-incorporated titanium oxide (TiO_2_) layers, using plasma electrolytic oxidation.	Bactericidal action against *E. coli.* The biological reaction to the phosphorus-, calcium-, and copper-containing layer has improved MG-63 osteoblastic cell integration, proliferation, and viability.
[144]	By using one-step micro-arc oxidation (MAO) technology, zinc and strontium were added to the surface coating of implants in different concentrations.	Bone marrow mesenchymal stem cells (BMSCs) can be effectively promoted to proliferate and differentiate when exposed to *S. aureus* and *P. gingivalis*; exhibits good antibacterial activity against these bacteria, and greater proliferation is seen in the cells on the coating with a higher strontium level.
